# Supplementary material for: Allele specific expression analysis identifies regulatory variation associated with stress-related genes in the Mexican highland maize landrace Palomero Toluqueño
Source: PeerJ. 2017 Aug 23;5:e3737. doi: 10.7717/peerj.3737 (PMC5572453; doi:10.7717/peerj.3737)
Supplement: Supplemental Information 1 [file peerj-05-3737-s001.pdf]

Input files:

- 11B-872-3.Ac4578.B73xEDMX-2233_palomero-1.fq
- 11B-872-3.Ac4578.B73xEDMX-2233_palomero-2.fq

Trim reads:

```
java -jar trimmomatic-0.32.jar PE -threads $PBS_NUM_PPN -phred33 \
[...]-1.fq [...]-2.fq   B73xPT_forward_paired.fq.gz B73xPT_forward_unpaired.fq.gz \
B73xPT_reverse_paired.fq.gz B73xPT_reverse_unpaired.fq.gz\
ILLUMINACLIP:TruSeq3-PE.fa:2:30:10 LEADING:3 TRAILING:3 SLIDINGWINDOW:4:15 MINLEN:36
```

Output: B73xPT_forward_paired.fq.gz, B73xPT_reverse_paired.fq.gz

---

Create bwa index:

```
bwa index -p B73_ref ../B73_GeneModels_longest.fa
```

Align trimmed reads:

```
bwa mem -t $PBS_NUM_PPN B73_ref B73xPT_forward_paired.fq.gz B73xPT_reverse_paired.fq.gz \
> B73xPT.sam
```

Output: B73xPT.sam

Convert sam to bam:

```
samtools view -b -@ $PBS_NUM_PPN -o B73xPT.bam B73xPT.sam
```

Output: B73xPT.bam

Sort bam:

```
samtools sort -@ $PBS_NUM_PPN -T temp -o B73xPT_sorted.bam B73xPT.bam
```

Output: B73xPT_sorted.bam

Remove duplicates:

```
samtools rmdup B73xPT_sorted.bam B73xPT_sorted_nodup.bam
```

Output: B73xPT_sorted_nodup.bam

Index sorted, no-duplicates bam:

```
samtools index B73xPT_sorted_nodup.bam
```

Output: B73xPT_sorted_nodup.bam.bai

Pileup:

```
samtools mpileup -g -Q 20 -q 30 -d 1000000 -f B73_GeneModels_longest.fa \
-o PT_mpileup.bcf B73xPT_sorted_nodup.bam
```

Output: PT_mpileup.bcf

Call SNPs:

```
bcftools call --threads $PBS_NUM_PPN -c -o PT_mpileup_call.vcf -O v PT_mpileup.bcf
```

Output: PT_mpileup_call.vcf

Filter SNPs:

```
vcfutils.pl varFilter -d100 PT_mpileup_call.vcf > PT_mpileup_call_flt.vcf
```

Output: PT_mpileup_call_flt.vcf

Compress vcf:

```
bgzip PT_mpileup_call_flt.vcf
```

Output: PT_mpileup_call.vcf.gz

Index compressed vcf:

```
tabix -p vcf PT_mpileup_call_flt.vcf.gz
```

Output: PT_mpileup_call.vcf.gz.tbi

Create consensus fasta:

```
bcftools consensus -f B73_GeneModels_longest.fa -o PT_ref.fasta \
PT_mpileup_call_flt.vcf.gz
```

Output: PT_ref.fasta

Change sequence headers:

```
perl -p -e "s/B73/PT/g" PT_ref.fasta > PT_GeneModels.fa
```

Output: PT_GeneModels.fa

Create pseudo-reference fasta:

```
cat B73_GeneModels_longest.fa PT_GeneModels.fa > F1_ref.fasta
```

Output: F1_ref.fasta

Create bowtie2 index:

```
bowtie2-build --threads $PBS_NUM_PPN --seed 99199 -o 2 F1_ref.fasta F1_ref
```

Output: a bowtie2 index named **F1_ref**

Align trimmed reads:

```
bowtie2 -a -I 100 -X 600 --rdg 6,5 --rfg 6,5 --score-min L,-.6,-.4 \
--no-discordant --no-mixed -p 20 -x ../index_bowtie2/F1_ref \
-1 ../fastq/B73xPT_forward_paired.fq.gz -2 ../fastq/B73xPT_reverse_paired.fq.gz \
-S F1_bowtie2.sam
```

Output: F1_bowtie2.sam

Convert sam to bam:

```
samtools view -h -b -@ $PBS_NUM_PPN -o F1_bowtie2.bam F1_bowtie2.sam
```

Output: F1_bowtie2.bam

---

n-sort bam:

```
samtools sort -@ $PBS_NUM_PPN -n -T temp -o F1_bowtie2_nsorted.bam F1_bowtie2.bam
```

Output: F1_bowtie2_nsorted.bam

Assign reads to transcripts:

```
express --no-update-check -H haplotype.txt -B 100 -o ./express_B100/ F1_ref.fasta F1_bowtie2_nsor
ted.bam
```

Output: results.xprs

Create table of B73 and PT effective counts for each transcript id:

```
#!/usr/bin/perl

$file_in = "results.xprs";
$file_out = "F1_counts.txt";

# create a hash of reads per B73 or PT transcript
$r = undef;
open ($r, "<", $file_in) or die $!;
<$r>; # .xprs header
while ($line = <$r>)
{
    chomp $line;
    next if ($line eq "");
    @a = split("\t", $line);
    undef $allele;
    undef $id;
    undef $tr;
    undef $counts;
    $id = $a[1];
    $counts = $a[7]; # the effective counts column
    #
    $id =~ /^(\w{2,3})_(.+)/;
    $allele = $1; # $allele is either "B73" or "PT"
    $tr = $2; # $tr is the transcript id
    $hash{$tr}{$allele} = $counts;
}
close $r;
#
# write table
$w = undef;
open ($w, ">", $file_out) or die $!;
print $w "tr\tB73\tPT\n";
foreach $tr (keys(%hash))
{
    print $w $tr;
    foreach $allele (sort(keys(%{$hash{$tr}})))
    {
        print $w "\t".$hash{$tr}{$allele};
    }
    print $w "\n";
}
close $w;
```

Output: F1_counts.txt

Identify transcripts with variants:

```
#!/usr/bin/perl

use Bio::SeqIO; # bioperl

# read B73 sequences
$file = "B73_GeneModels_longest.fa";
%b73 = ();
$seqio_obj = Bio::SeqIO->new(-file => $file, -format => "fasta" );
while ($seq_obj = $seqio_obj->next_seq)
```

```
{
    undef $id;
    undef $tr;
    undef $seq;
    $id = $seq_obj->primary_id;
    $seq = $seq_obj->seq;
    $id =~ /\w{2,3}_(.+)/;
    $tr = $1;
    $b73{$tr} = $seq;
}
print scalar(keys(%b73))." B73 sequences\n";
#
# read PT sequences
$file = "PT_GeneModels.fa";
%pt = ();
$seqio_obj = Bio::SeqIO->new(-file => $file, -format => "fasta" );
while ($seq_obj = $seqio_obj->next_seq)
{
    undef $id;
    undef $tr;
    undef $seq;
    $id = $seq_obj->primary_id;
    $seq = $seq_obj->seq;
    $id =~ /\w{2,3}_(.+)/;
    $tr = $1;
    $pt{$tr} = $seq;
}
print scalar(keys(%pt))." PT sequences\n"; # should be the same as B73
#
# get transcript and locus id when sequences don't match
%diff_tr = %diff_locus = ();
foreach $tr (keys(%b73))
{
    if ($b73{$tr} ne $pt{$tr})
    {
        undef $locus;
        $locus = $tr;
        $locus =~ s/_FGT/_FG/;
        $locus =~ s/_T\d+$//;
        $diff_tr{$tr} = 1;
        $diff_locus{$locus} = 1;
    }
}
#
# write transcripts and loci lists
$w = undef;
open ($w, ">", "SNP_tr.txt") or die $!;
    print $w join("\n", sort(keys(%diff_tr)));
close $w;
#
$w = undef;
open ($w, ">", "SNP_loci.txt") or die $!;
    print $w join("\n", sort(keys(%diff_locus)));
close $w;
```

Output: SNP_tr.txt (and SNP_loci.txt for gene enrichment analyses)

Identify transcripts with ASE (R script):

```
library(dplyr)

rm(list=ls())

# get counts table
F1_counts_full <- read.delim("F1_counts.txt", as.is=TRUE)

# locus column
F1_counts_full$locus <- sub("_FGT", "_FG", F1_counts_full$tr)
F1_counts_full$locus <- sub("_T\\d{2}", "", F1_counts_full$locus)

# insert chr, start and stop from gff3 file
# ftp://ftp.ensemblgenomes.org/pub/plants/release-22/gff3/zea_mays/
# read gff3 and get transcript names
file_gff <- "Zea_mays.AGPv3.22.gff3"
gff <- read.delim(file_gff, header=FALSE, as.is=TRUE)
gff <- gff[gff$V3 == "transcript", ]
getid <- function(z)
{
    x <- gff[z, "V9"]
    m <- regexpr("ID=.+?;", x)
    regmatches(x, m)
}
gff$tr <- sapply(1:nrow(gff), getid)

# clean trancript names
gff$tr <- sub("ID=transcript:", "", gff$tr)
gff$tr <- sub(";", "", gff$tr)

# insert chr, start and stop
names(gff)[names(gff) == "V1"] <- "chr"
names(gff)[names(gff) == "V4"] <- "start"
names(gff)[names(gff) == "V5"] <- "stop"
F1_counts_full <- left_join(F1_counts_full, gff[, c("tr", "chr", "start", "stop")], by="tr")

# ratio and fold change
F1_counts_full$ratio <- (F1_counts_full$PT/F1_counts_full$B73)
F1_counts_full$log2_fc <- log((F1_counts_full$PT/F1_counts_full$B73), 2)

# identify transcripts with ASE
F1_counts <- F1_counts_full
# for F1_counts keep only polymorphic transcripts (i.e. transcripts with SNPs)
file_polymorphs <- "SNP_tr.txt"
polymorphs <- scan(file_polymorphs, what="character", sep="\n")
F1_counts <- F1_counts[F1_counts$tr %in% polymorphs, ]

# no transcripts with 0 reads in B73
F1_counts <- filter(F1_counts, B73 != 0)

# chisq test
F1_counts$chi_sq <- sapply (1:nrow(F1_counts), function(z) {
      chisq.test(c(F1_counts[z, "B73"], F1_counts[z, "PT"]),p=c(.5, .5))$statistic
      })

# p-value
F1_counts$p_value <- sapply (1:nrow(F1_counts), function(z) {
      chisq.test(c(F1_counts[z, "B73"], F1_counts[z, "PT"]), p=c(.5, .5))$p.value
      })
```

```
# FDR, Bonferroni and Holm
F1_counts$fdr <- p.adjust(F1_counts$p_value, method="BH")
F1_counts$bon <- p.adjust(F1_counts$p_value, method="bonferroni")
F1_counts$holm <- p.adjust(F1_counts$p_value, method="holm")

# ASE and ASE_call tag
get_ASE <- function(z)
{
    if (F1_counts[z, "bon"] < 0.05 & F1_counts[z, "log2_fc"] >= 1)
    {
        1
    }
    else if (F1_counts[z, "bon"] < 0.05 & F1_counts[z, "log2_fc"] <= -1)
    {
        -1
    }
    else
    {
        0
    }
}
F1_counts$ASE_call <- sapply(1:nrow(F1_counts), get_ASE)
F1_counts$ASE <- abs(F1_counts$ASE_call)

# write ASE lists
write(unique(F1_counts[F1_counts$ASE == 1, "locus"]), "ASE_loci.txt", sep="\n")
write(unique(F1_counts[F1_counts$ASE == 1, "tr"]), "ASE_tr.txt", sep="\n")

# write F1 table
F1_counts_write <- left_join(F1_counts_full[, c("tr", "locus", "chr", "start", "stop", \
"B73", "PT", "ratio", "log2_fc")], F1_counts[, c("tr", "chi_sq", "p_value", "fdr", "bon", \
"holm", "ASE", "ASE_call")], by="tr")
write.table(F1_counts_write, "F1_counts_chisq.tsv", quote=FALSE, row.names=FALSE, sep="\t")
```

Output: F1_counts_chisq.tsv table of transcripts with ASE.
